# Development of highly discriminatory SCoT- and CBDP-based SCAR fingerprint for authentication of Indian senna (*Senna alexandrina* Mill.) formerly *Cassia angustifolia* Vahl.)

**DOI:** 10.3389/fpls.2024.1424665

**Published:** 2024-07-04

**Authors:** Sarika Chouksey, Mohd Ashraf Ashfaq, Pushkar Kaira, Sabnam Farhat, Maneesha Pandey, Ch. Anil Kumar, Rama Reddy Nagaraja Reddy

**Affiliations:** ^1^ Biochemistry Discipline, School of Sciences, Indira Gandhi National Open University, New Delhi, India; ^2^ Department of Botany, School of Chemical and Life Sciences, Jamia Hamdard, New Delhi, India; ^3^ Biotechnology, Indian Council of Agricultural Research (ICAR)- Indian Institute of Oilseeds Research, Hyderabad, India; ^4^ Plant Breeding, Indian Council of Agricultural Research-Directorate of Medicinal and Aromatic Plants Research (ICAR-DMAPR), Boriavi Anand, Gujarat, India

**Keywords:** *Senna alexandrina* Mill., Indian senna, SCOT, CBDP, SCAR, adulterant species

## Abstract

**Introduction:**

Indian senna (*Senna alexandrina* Mill.) (formerly *Cassia angustifolia* Vahl.) is an important medicinal plant of the family Fabaceae. The leaves and pods of Indian senna yield sennosides and rhein-based laxative. Adulteration of Indian senna is a serious issue as with most of the medicinal plants used in the Indian systems of traditional medicine. The bulk of dried leaves and pods of morphologically related species, such as *Cassia fistula*, *Senna occidentalis*, *Senna sophera*, and *Senna tora*, is usually mixed with those of the Indian senna, and the admixture is used in laxative-based formulations. The present investigation is a modest attempt at developing species-specific start codon targeted (SCoT) polymorphism- and CAAT-box-derived polymorphism (CBDP)-based sequence-characterized amplified region (SCAR) markers for the identification and authentication of Indian senna and four adulterant species (*C. fistula*, *S. occidentalis*, *S. sophera*, and *S. tora* species).

**Methods:**

In this study, genomic DNA extracted from 44 accessions of Indian senna and four adulterant species was subjected to SCoT and CBDP PCR. The polymorphic amplicons were identified, eluted, ligated, and transformed into *Escherichia coli* DH5 α strain. PCR, restriction analysis, and DNA sequencing confirmed the transformed recombinant plasmid clones.

**Results:**

Post-sequencing, the sequence of the primary SCoT and CBDP primers was analyzed and extended into the unique signature sequence of the concerned accessions. This resulted in development of one SCoT-44- and two CBDP-25-based SCARs. SCoT-44 SCAR produced a signature amplicon of 287 bp for accession DCA120, and CBDP-25 SCAR yielded signature amplicons of 575 and 345 bp for accessions DCA13 and DCA119, respectively. The developed SCAR markers were validated across 48 samples (44 accessions of Indian senna and 4 adulterant species) and produced distinct amplicons in Indian senna only, while no such amplicon was observed in the other four adulterant species.

**Discussion:**

The information generated using these markers have been faithfully converted to single-locus, unequivocal, highly reproducible, and informative sequence-based SCAR markers. These markers will enable discrimination of individual plants on the basis of unique sequence-specific amplicons, which could be used as diagnostic markers to settle issues pertaining to the true identity of Indian senna.

## 1 Introduction

*Senna alexandrina* Mill. (commonly known as Indian senna), an important member of the family Fabaceae (sub-family Caesalpiniaceae), is a major natural laxative-yielding medicinal plant (Reddy et al., 2015). A native to Egypt, Sudan, Nigeria, North Africa, India, Pakistan, China, and Sinai, the Indian senna is an erect perennial subshrub bearing pinnately compound leaves with lanceolate, glabrous green leaflets. The stem bears drooping branches with racemose inflorescence. The plant abounds in more than 28 bioactive compounds. The leaves and pods are the economically important parts of Indian senna and are a good source of anthraquinone-based sennosides A, B, C, and D and rhein. Sennosides, largely found in leaves (2%–3%) and pods (3%–4%) of Indian senna, are diglucosides of sennidins (Chadha and Gupta, 1995). Additionally, the roots contain rhein, chrysophanol, emodin, and aloe–emodin (Ramchander et al., 2017).

Adulteration and substitution are issues of concern in the herbal industry necessitating authentication and standardization of medicinal plants. Approaches based on powder microscopy, biochemistry, and molecular biology have been used to identify and authenticate Indian senna. Classical light microscopy coupled with scanning electron microscopy, fluorescence microscopy, and chemo profiling were employed toward establishing quality control for adulteration of Indian senna (Sultana, 2012; Shaheen et al., 2019). Authenticity of a 200-year-old “Extractum Sennae” was confirmed by reversed-phase high-performance liquid chromatography (RP-HPLC) and electrospray ionization mass spectrometry (ESI-MS*^n^*) (Nesmerak et al., 2020). Identification and authentication of medicinal plants using molecular markers is indispensable as it seeks to provide unmatched identity of the species of interest (Joseph et al., 2014). This is documented by authentication of genuine Indian senna from the adulterant species using OPC-17 and OPC-18 random amplified polymorphic DNA (RAPD) markers (Khan et al., 2011). Patent for sequence-characterized amplified region (SCAR) primer-based on RAPD has been filed for award of the same for authentication of true-to-type Indian senna (https://www.quickcompany.in/patents/scar-primers-and-a-kit-for-the-authentication-of-unani-drug-senna-acutifolia-cassia-angustifolia-from-its#documents). DNA barcoding coupled with high-resolution melting (HRM) curve analysis has been used for undisputed authentication of Indian senna (Mishra et al., 2018).

A PCR-based gene-targeted functional marker, start codon targeted (SCoT) polymorphism employs a single 18-mer-long primer (which behaves both as forward and reverse primer) and is based on short-conserved region flanking the start codon (ATG) in plant genes (Collard and Mackill, 2009). SCoT marker correlates with functional genes and associated characteristics without requiring sequence information (Mulpuri et al., 2013). It generates a better fingerprint than RAPD, inter-simple sequence repeat (ISSR), and other multi-locus markers.

SCoT markers were extrapolated for authentication in rice (Collard and Mackill, 2009); *Plantago* (Rahimi et al., 2018), cowpea (Igwe et al., 2017), *Tritordeum bergrothii* L (Cabo et al., 2014), and soybean cultivars (Rayan and Osman, 2019). SCoT markers proved to be superior over RAPD and ISSR in terms of diversity index, marker index, and resolving power. SCoT yielded more polymorphism and scorable amplicons compared to RAPD (Gorji et al., 2011). Further, Amom et al. (2020) have credited SCoT with more information and higher discriminating power than RAPD and ISSR.

Another functional molecular marker, CAAT-box-derived polymorphism (CBDP), based on polymorphism due to the promoter region of genes, utilizing primers designed from promoter consensus CAAT-box region (Singh et al., 2014), has been employed in the present study to authenticate Indian senna. The CAAT-box is an essential motif in transcription and has a unique conserved nucleotide pattern with the consensus sequence GGCCAATCT. It is roughly 80 bp upstream of the start codon of eukaryotic genes. CBDP markers have been used for identification of genetic diversity in several crops such as cotton and linseed cultivars (Singh et al., 2014), jojoba genotypes (Heikrujam et al., 2015), and *Andrographis paniculata* (Tiwari et al., 2016). Studies that employed both SCoT and CBDP markers include examination of the genetic diversity present in various *Aegilops* species (Pour-Aboughadareh et al., 2019) and fidelity of the clones produced by micro-propagation of *Brassica racemosa* (Sharma et al., 2019).

SCAR is a polymorphic region of a known sequence, which is invariably an extension of sequence of the primary marker system. Initially, SCAR was developed for isolating downy mildew resistance genes in lettuce (Paran and Michelmore, 1993). These are mono-locus, usually co-dominant PCR-based markers that require two sequence-specific primers. SCAR may be developed from RAPD (Paran and Michelmore, 1993), amplified fragment length polymorphism (AFLP) (Liang et al., 2011), ISSR (Ghosh et al., 2011), and SCoT (Mulpuri et al., 2013). Hence, the results with these markers are more reliable and reproducible. SCoT based SCAR makers were developed to distinguish toxic and non-toxic accessions of *Jatropha curcas* L (Mulpuri et al., 2013). and authenticate *Taxus media* (Hao et al., 2018) and *Physalis* (Solanaceae) species (Feng et al., 2018).

With this background, the objective of the study was to develop reliable SCoT- and CBDP-based SCAR markers for the authentication of Indian senna.

## 2 Materials and methods

### 2.1 Plant material and DNA extraction

The panel of plant material used in the study included 48 samples comprising 44 accessions of Indian senna (kindly provided by RNR, ICAR-DMAPR, Anand, Gujarat, India) and 4 adulterant species (*C. fistula*, *S. occidentalis*, *S. sophera*, and *S. tora*) (**Table 1**). Fresh young leaves from germinated seedlings of Indian senna and four adulterant *Senna* species were used for DNA isolation using the CTAB method (Doyle, 1991) with modification. The quality and integrity of the isolated genomic DNA was checked on 1% agarose (w/v) (Hi media MB grade) gel electrophoresis and documented by comparing it to the fluorescence yield of the standards—uncut, λ DNA (50 and 100 ng). DNA samples were appropriately diluted to 50 ng/µl in TE buffer and used for PCR amplification.

**Table 1:** Voucher number information of 44 accessions of Indian senna and 4 adulterant species used in the development of SCoT and CBDP based SCARs.

S.No.	Species	Voucher No.	District	State	Longitude	Latitude
1.	*Senna alexandrina* Mill.	DCA- 09	Jodhpur	Rajasthan	73°04.00’E	26°11.00’ N
2.		DCA- 10	Jodhpur	Rajasthan	73°04.00’E	26°11.00’ N
3.		DCA-12	Jodhpur	Rajasthan	72°40.00’E	26°18.00’ N
4.		DCA-13	Jodhpur	Rajasthan	72°14.00’E	26°39.00’ N
5.		DCA-25	Barmer	Rajasthan	71°18.00’E	25°45.00’ N
6.		DCA-39	Anand	Gujarat	72° 97.00’E	22 °53.00’N
7.		DCA-42	Killikulam	Tamil Nadu	77 °,85.00’E	8° 70.00’N
8.		DCA-45	Jodhpur	Rajasthan	72 °,99.00’E	26° 26.00’N
9.		DCA-47	Jodhpur	Rajasthan	72°44.00’E	26°18.00’N
10.		DCA-52	Jodhpur	Rajasthan	73°56.00’E	25°40.00’ N
11.		DCA-56	Jodhpur	Rajasthan	72°29.00’E	27°01.00’ N
12.		DCA-60	Jodhpur	Rajasthan	72°19.00’E	26°35.00’ N
13.		DCA-70	Pali	Rajasthan	73°26.00’E	25°49.00’ N
14.		DCA-75	Jodhpur	Rajasthan	73°11.70’E	26°32.20’ N
15.		DCA-85	Surendranagar	Gujarat	71°10.00’E	22°25.12’ N
16.		DCA-88	Bhuj	Gujarat	70°05.30’E	23°08.19’ N
17.		DCA-89	Bhuj	Gujarat	69°54.17’E	23°15.50’ N
18.		DCA-90	Bhuj	Gujarat	70°00.72’E	23°17.26’ N
19.		DCA-96	Virudhunagar	Tamil Nadu	78° 06.37’E	9° 22.30’N
20.		DCA-97	Virudhunagar	Tamil Nadu	78° 00.11’E	9° 08.76’ N
21.		DCA-100	Tirunelveli	Tamil Nadu	77° 35.76’E	8° 21.18’N
22.		DCA-101	Tirunelveli	Tamil Nadu	77° 36.35’E	8° 23.55’N
23.		DCA-103	Tuticorin	Tamil Nadu	77° 52.01’E	8° 44.76’N
24.		DCA-105	Tuticorin	Tamil Nadu	77° 42.37’E	8° 48.54’N
25.		DCA-107	Tuticorin	Tamil Nadu	78° 17.22’E	9° 03.79’N
26.		DCA-108	Ramanathapuram	Tamil Nadu	79° 05.57’E	9° 16.59’N
27.		DCA-109	Virudhunagar	Tamil Nadu	77° 58.01’E	9° 40.44’N
28.		DCA-112	Virudhunagar	Tamil Nadu	77° 57.53’E	9° 34.80’N
29.		DCA-114	Madurai	Tamil Nadu	77° 51.26’E	9° 45.60’N
30.		DCA-115	Madurai	Tamil Nadu	77 ° 51.26’E	9° 45.60’N
31.		DCA-117	Madurai	Tamil Nadu	77° 52.42’E	9° 44.77’N
32.		DCA-119	Madurai	Tamil Nadu	77 ° 47.70’E	9° 42.26’N
33.		DCA-120	Tuticorin	Tamil Nadu	77° 52.01’E	8° 44.76’N
34.		DCA-121	Anand	Gujarat	72 ° 93.04’E	22° 59.91’N
35.		DCA-123	Bikaner	Rajasthan	73° 12.45’E	28° 02.53’N
36.		DCA-127	Bikaner	Rajasthan	73° 10.24’E	28° 02.83’N
37.		DCA-128	Bikaner	Rajasthan	73° 05.77’E	27° 59.17’N
38.		DCA-129	Bikaner	Rajasthan	73° 03.57’E	27° 58.38’N
39.		DCA-130	Bikaner	Rajasthan	72° 59.91’E	27° 55.79’N
40.		DCA-131	Bikaner	Rajasthan	72° 59.04’E	27° 54.71’N
41.		DCA-147	Jaisalmer	Rajasthan	71° 53.86’E	26° 56.32’N
42.		DCA-150	Jaisalmer	Rajasthan	71° 53.86’E	26° 56.32’N
43.		DCA-155	Jodhpur	Rajasthan	72° 29.56’E	27° 08.61’N
44.		DCA-156	Jodhpur	Rajasthan	72° 32.54’E	27° 08.69’N
45.	*Cassia fistula*	F	South Delhi	Delhi	77°15’6.264 E.	28°31’48.32’’N
46.	*Senna occidentalis* (L.)	O	South Delhi	Delhi	77°15’6.264 E	28°31’48.324’’N
47.	*Senna sophera* (L.)	S	South Delhi	Delhi	77°15’6.264 E.	28°31’48.324’’ N
48.	*Senna tora* (L.)	T	South Delhi	Delhi	77° 15’ 6.264 E.	28°31’48.324’’ N

### 2.2 Screening and selection of SCoT and CBDP primers

Based on the available primer sequences in the public domain, 16 SCoT and 25 CBDP (Singh et al., 2014) primers were custom synthesized from BioServe Biotechnologies (India) Pvt. Ltd. The primers that yielded robust SCoT and CBDP profiles were then selected, and the entire panel of 48 DNA samples (44 accessions of Indian senna + 4 adulterant species) (**Table 1**) were then subjected to SCoT-PCR and CBDP-PCR.

### 2.3 PCR amplification with selected SCoT and CBDP primers

The PCR reaction was carried out in 15-µl reaction volume containing 1× PCR buffer, 50 ng of genomic DNA as template, 1.5 mM of MgCl_2_, 160 µM of dNTPs, 1.0 µM of SCoT and CBDP primers, and 0.5 U of *Taq* DNA polymerase. PCR amplifications were performed with the initial denaturation at 94°C for 4 min followed by 45 cycles of denaturation at 94°C for 1 min, annealing at 50°C for 1 min, and extension at 72°C for 2 min with a final extension at 72°C for 10 min. The PCR products were separated by electrophoresis in 3.5% (w/v) agarose gel for 2–3 h at 100 V, and 100 bp was loaded as the standard-size ladder and profiled using a gel documentation system.

### 2.4 SCAR marker development

#### 2.4.1 Conversion of SCoT and CBDP amplicons into SCAR markers

SCoT-44 (5′-ACGACATGGCGACCCACA-3′) and CBDP-25 primers (5′-CTGAGCACGATCCAATGT-3′) were subjected to PCR for cloning (**Tables 2** and **3**). A 3.5% (w/v) agarose gel was run for 2–3 h at 100 V after PCR, and the desired amplicons were cut for further elution.

**Table 2:** List of 16 SCoT primers used in the study.

S.No.	Primer	Primer Sequence (5´-3´)	Length (bases)	Tm (°C)	GC Content (%)
1.	SCoT 8	ACCATGGCTACCACCGCA	18	53	61.11
2.	SCoT 21	CCATGGCTACCACCGCCT	18	55	66.66
3.	SCoT 27	ACAATGGCTACCACTGCC	18	50	55.55
4.	SCoT 42	ACCATGGCTACCACCGGC	18	55	66.66
5.	SCoT 43	ACCATGGCTACCACCGCC	18	55	66.66
6.	SCoT 44	ACGACATGGCGACCCACA	18	53	61.11
7.	SCoT 66	ACAATGGCTACCACTAGC	18	48	50.00
8.	SCoT 7	ACAATGGCTACCACTGAC	18	48	50.00
9.	SCoT 10	ACAATGGCTACCACCAGC	18	50	55.55
10.	SCoT 11	ACAATGGCTACCACTACC	18	48	50.00
11.	SCoT 14	ACCATGGCTACCAGCGCG	18	55	66.66
12.	SCoT 24	CCATGGCTACCACCGCAG	18	55	66.66
13.	SCoT 28	CAACAATGGCTACCACCA	18	48	50.00
14.	SCoT 32	CAACAATGGCTACCACGC	18	50	55.55
15.	SCoT 35	AACCATGGCTACCACCAC	18	50	55.55
16.	SCoT 46	ACCATGGCTACCACCGCC	18	55	66.66

**Table 3:** List of 25 CBDP primers used in the study.

S.No.	Primer	Primer Sequence (5´-3´)	Length (bases)	Tm (°C)	GC Content (%)
1.	CBDP 1	TGAGCACGATCCAATAGC	18	48	50.00
2.	CBDP 2	TGAGCACGATCCAATAAT	18	43	38.88
3.	CBDP 3	TGAGCACGATCCAATACC	18	48	50.00
4.	CBDP 4	TGAGCACGATCCAATAAG	18	46	44.44
5.	CBDP 5	TGAGCACGATCCAATCTA	18	46	44.44
6.	CBDP 6	TGAGCACGATCCAATCAG	18	48	50.00
7.	CBDP 7	TGAGCACGATCCAATCGA	18	48	50.00
8.	CBDP 8	TGAGCACGATCCAATCGG	18	50	55.55
9.	CBDP 9	TGAGCACGATCCAATGAT	18	46	44.44
10.	CBDP 10	TGAGCACGATCCAATGTT	18	46	44.44
11.	CBDP 11	TGAGCACGATCCAATTGC	18	48	50.00
12.	CBDP 12	TGAGCACGATCCAATATA	18	43	38.88
13.	CBDP 13	TGAGCACGATCCAATGAG	18	48	50.00
14.	CBDP 14	TGAGCACGATCCAATGCG	18	50	55.55
15	CBDP 15	TGAGCACGATCCAATTGA	18	46	44.44
16.	CBDP 16	TGAGCACGATCCAATTCA	18	46	44.44
17.	CBDP 17	TGAGCACGATCCAATTTG	18	46	44.44
18.	CBDP 18	CTGAGCACGATCCAATAG	18	48	50.00
19.	CBDP 19	CTGAGCACGATCCAATAC	18	48	50.00
20.	CBDP 20	CTGAGCACGATCCAATAT	18	46	44.44
21.	CBDP 21	CTGAGCACGATCCAATCA	18	46	50.00
22.	CBDP 22	CTGAGCACGATCCAATCG	18	50	55.55
23.	CBDP 23	CTGAGCACGATCCAATGG	18	50	55.55
24.	CBDP 24	CTGAGCACGATCCAATGA	18	48	50.00
25.	CBDP 25	CTGAGCACGATCCAATGT	18	48	50.00

#### 2.4.2 Elution, ligation, cloning, and sequencing of amplicons

The DNA from the respective SCoT-44 and CBDP-25 gel profiles were eluted by following the manufacturer’s instructions as given in the QIAgen Gel Extraction kit. The eluted DNA was then subjected to electrophoresis on 1.2% (w/v) agarose gel to know the integrity and quantity of the eluted DNA. The eluted polymorphic amplicons were subjected to T/A cloning. The pGEM^®^-T Easy Vector (Promega, USA) has been used for cloning the eluted polymorphic fragments. The ligated product was transformed in competent (CaCl_2_ treated) *E. coli* DH5α following the heat-shock method. Blue-white screening was undertaken to select the recombinant from the non-recombinant colonies. Positive clones of Indian senna accessions were identified using PCR with M13 forward and reverse primers and restriction digestion of recombinant clones using *Not* I. The positive clones were then sequenced.

#### 2.4.3 Analysis of DNA sequences using BLAST

The online site for basic local alignment search tool (BLAST) (https://blast.ncbi.nlm.nih.gov/Blast.cgi) was searched for exploring similarity of the obtained sequences with any reported sequences. Separation of the vector sequences were undertaken, and the trimmed sequences were subjected to BLAST analysis.

#### 2.4.4 Designing of SCAR primers

The trimmed sequences were then fed to online primer design software, OligoCalc (Kibbe, 2007). A primer pair (SCAR forward and reverse) was then designed for each of the sequences by extending the length of the original primer of the primary marker systems (SCoT and CBDP) into the sequence of the accession of interest.

#### 2.4.5 Validation of SCAR markers

For validation of the designed SCoT–SCAR and CBDP–SCAR, PCR was carried out in a total volume of 15 µl, which included 1× PCR buffer with 1.5 mM of MgCl_2_, 100 µM of dNTPs, 1 µM of forward primer, 1 µM of reverse primer, 50 ng of DNA, 0.5 U of *Taq* DNA polymerase, and MilliQ H_2_O to make up the reaction volume. The amplification was performed by following a PCR thermal profile: 94°C for 3 min followed by 35 cycles of 94°C for 1 min, annealing (SCoT PCR: 64°C; CBDP PCR: 64.5°C) for 45 s, 72°C for 45 s, and a final extension at 72°C for 10 min. The SCAR markers were validated across 44 accessions and 4 adulterant species. PCR products were separated in 2% (w/v) agarose gel and electrophoresed for 1–2 h at 100 V and documented using gel documentation system.

## 3 Results

### 3.1 Identification of polymorphic amplicons by screening of SCoT and CBDP primers

**Figure 1:**
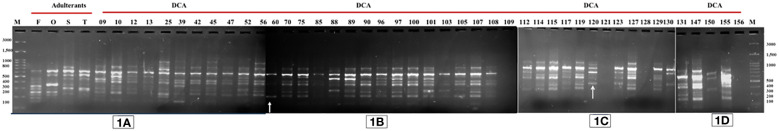
PCR profile of 48 samples with SCoT-44 primer. Lane M—100-bp DNA ladder. **(A)** SCoT PCR of four adulterant species (*Cassia fistula* (F), *Senna occidentalis* (O), *Senna sophera* (S), and *Senna tora* (T)) and accessions of Indian senna (DCA9 to DCA56); **(B)** (DCA60 to DCA109) accessions; **(C)** (DCA112 to DCA130) accessions; **(D)** (DCA131 to DCA156) accessions of Indian senna. Polymorphic amplicons were obtained in DCA60 and DCA120 accessions of Indian senna. Arrows represent polymorphic amplicons in DCA60 and DCA120 accessions of Indian senna.

**Figure 2:**
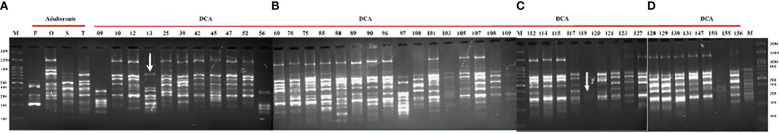
PCR profile of 48 samples with CBDP-25 primer. Lane M—100-bp DNA ladder. **(A)** CBDP PCR of four adulterant species (*Cassia fistula* (F), *Senna occidentalis* (O), *Senna sophera* (S), and *Senna tora* (T)) and accessions of Indian senna (DCA9 to DCA56); **(B)** (DCA60 to DCA109) accessions; **(C)** (DCA112 to DCA127) accessions; **(D)** (DCA128 to DCA156) accessions of Indian senna. Polymorphic amplicons were obtained in DCA13 and DCA119 accessions of Indian senna. Arrows represent polymorphic amplicons in DCA13 and DCA119 accessions of Indian senna.

The genomic DNA extracted from all 48 samples underwent initial primer screening. From a pool of 16 SCoT and 25 CBDP primers exhibiting distinct and consistent polymorphism, one SCoT-44 and one CBDP-25 primer were chosen for subsequent analysis across all samples. SCoT-44 and CBDP-25 primers (**Table 4**) were selected for PCR amplification of 48 samples. The criterion for choosing SCoT-44 and CBDP-25 primers out of 16 SCoT and 25 CBDP primers was that not all primers yielded robust banding pattern across all the 48 samples tested. Only those primers that gave at least one band across all the 48 samples were chosen, and thus, SCoT-44 and CBDP-25 primers were selected. The amplified loci were between 100 and 3,000 bp in size. SCoT-44 (**Figures 1A–D**) yielded polymorphic amplicons with DCA60 (**Figures 1A, B**), and DCA120 (**Figure 1C**) and CBDP-25 (**Figures 2A–D**) gave polymorphic amplicons with accessions DCA13 (**Figure 2A**) and DCA119 (**Figure 2C**). SCoT-44 and CAAT-25 primers generated amplicons that were chosen as potential Indian senna-specific markers.

**Table 4:** List of SCoT and CBDP primers chosen for development of SCARs.

Primer Name	Primer Sequence (5’-3’)	Length (bases)	Annealing temperature (°C)	Total No. of amplicons obtained (Across 48 samples)	Total No. of Monomorphic amplicons (Across 48 samples)	No. of Polymorphic amplicons (Across 48 samples)
SCoT-44	ACGACATGGCGACCCACA	18	50	329	327	2
CBDP-25	CTGAGCACGATCCAATGT	18	50	407	405	2

### 3.2 Cloning and sequencing of polymorphic amplicons

**Figure 3:**
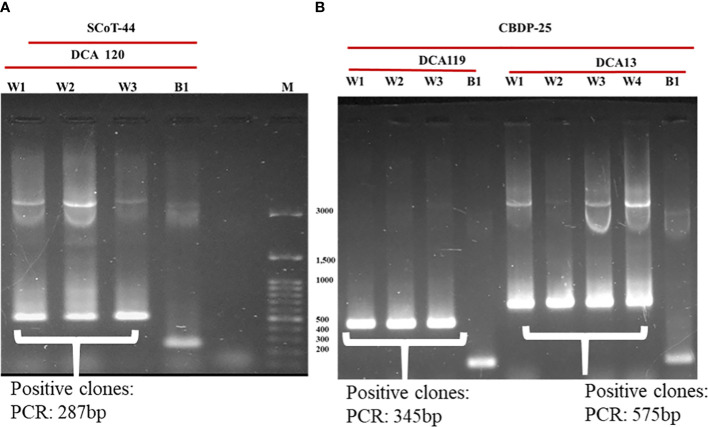
Confirmation of cloning of polymorphic amplicons obtained from SCoT-44 and CBDP-25 by PCR with M13 forward and reverse primers. **(A)** Recombinant clones (W1–W3); non-recombinant clone (B1) of DCA120; Lane M—100-bp DNA ladder; **(B)** Recombinant clones (W1–W3); non-recombinant clone (B1) of DCA119; recombinant clones (W1–W4); non-recombinant clone (B1) of DCA13.

**Figure 4:**
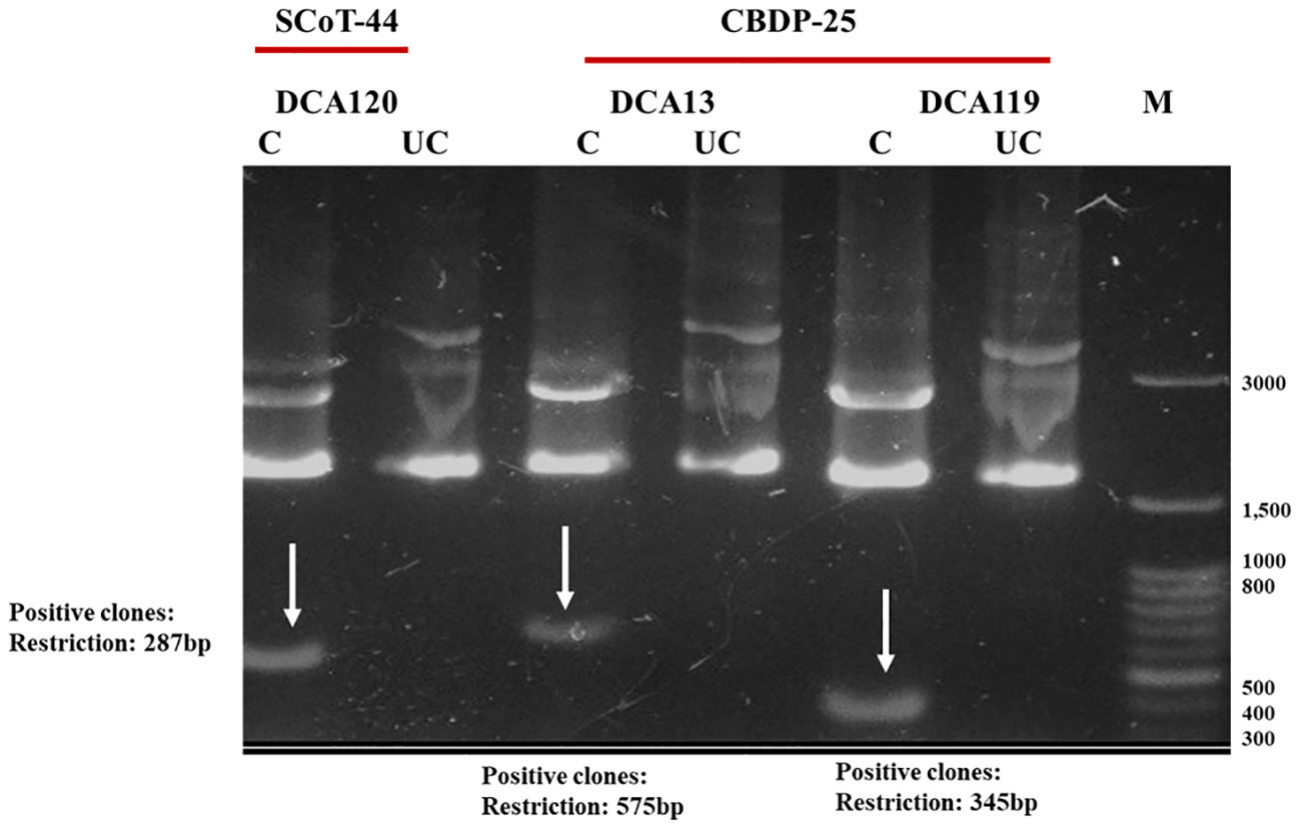
Restriction analysis of recombinant clones of SCoT-44 (DCA120 accession of Indian senna) and CBDP-25 (DCA13 and DCA119 accessions of Indian senna) using *Not* I; Lane M—100-bp DNA ladder; C, cut/restricted; UC, uncut/unrestricted.

**Figure 5:**
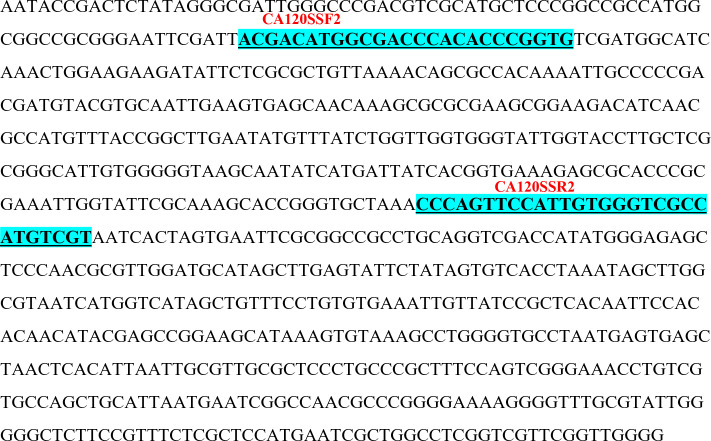
Nucleotide sequence (287 bases) (GenBank accession No. OR060948) of SCoT-44 SCAR specific for Indian senna accession DCA120. The underlined sequences indicate newly developed SCoT-44 based SCAR forward and reverse primers (CA120SSF2/CA120SSR2).

**Figure 6:**
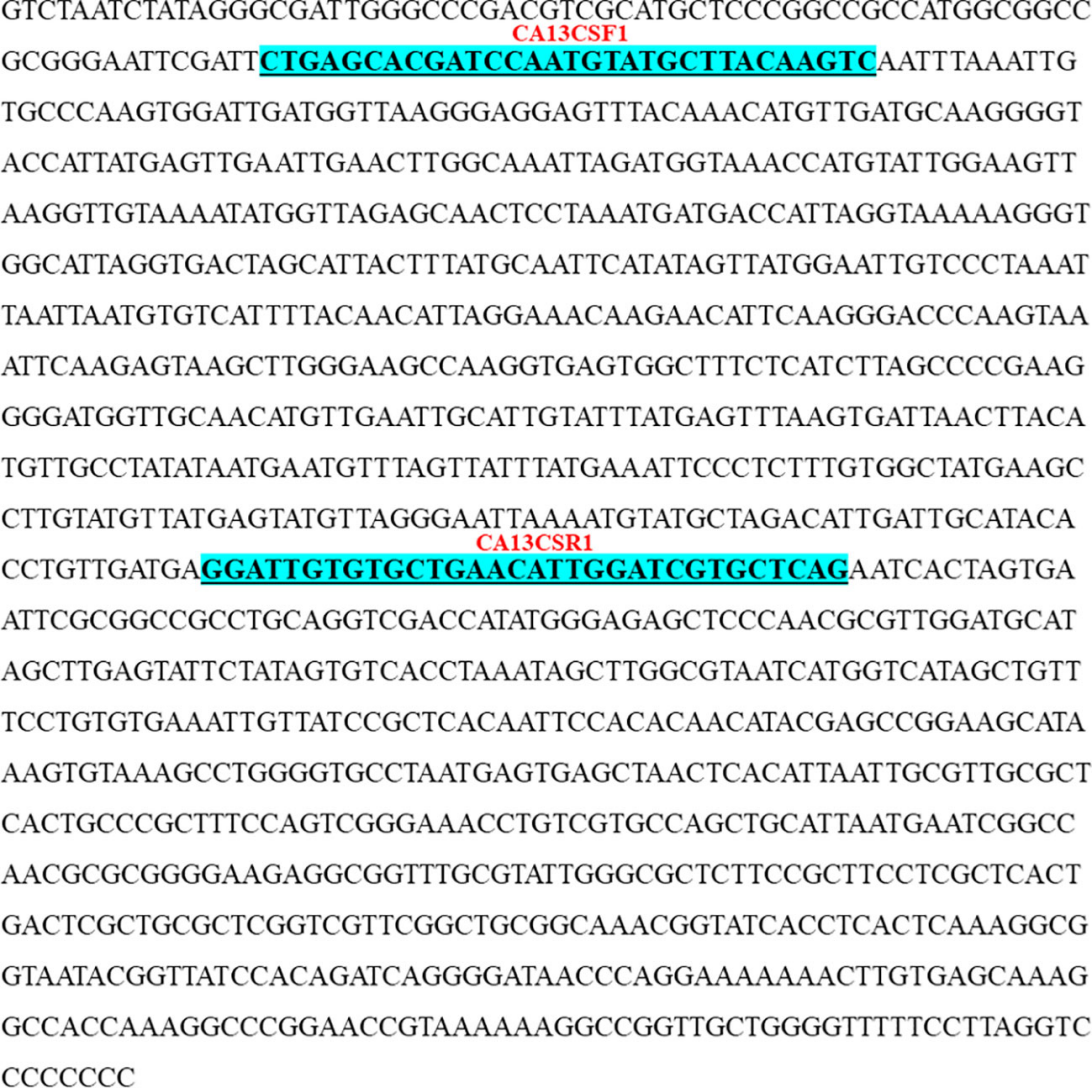
Nucleotide sequence (575 bases) (GenBank accession No. OR060949) of CBDP-25 SCAR for DCA13. The underlined sequences indicate newly developed CBDP-25 based SCAR forward and reverse primers (CA13CSF1/CA13CSR1).

**Figure 7:**
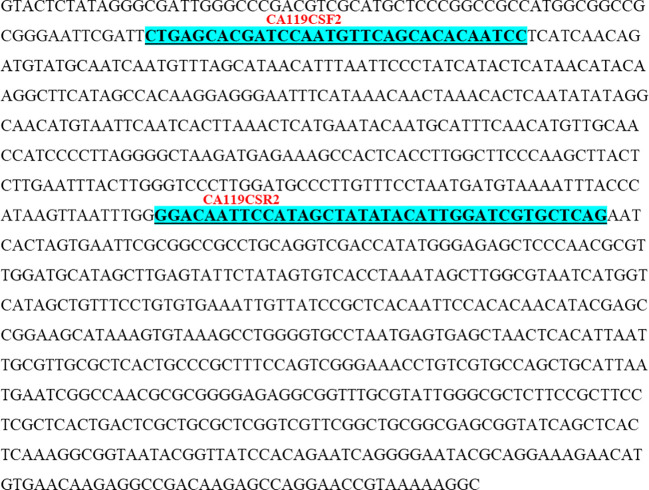
Nucleotide sequence (345 bases) (GenBank accession No. OR060950) of CBDP-25 SCAR specific for DCA119. The underlined sequences indicate newly developed CBDP-25 based SCAR forward and reverse primers (CA119CSF2/CA119CSR2).

Identified polymorphic amplicons were cut out from the gel and eluted using the Qiagen Gel Extraction kit. The amplicons were then cloned in Promega pGEM-T Easy vector. The positive clones were identified initially by blue-white screening. Plasmid DNA from the putative transformed recombinant clones was isolated and subjected to PCR with M13 forward and reverse primers (**Figures 3A, B**) and restriction digestion (**Figure 4**) using *Not* I. Restriction of the putative recombinant clones with *Not* I adds 22 bp to the size of the amplicon. One positive recombinant clone of SCoT-44 (DCA 120) and two clones of CBDP-25 (DCA13; DCA119) were then sequenced. Post-sequencing, the sequences were subjected to BLAST analysis. No significant similarities were found with any other sequences in the GenBank database. These sequences are unique to the Indian senna species and did not give any significant hit to any known sequences in the public domain. With SCoT-44, a signature amplicon of 287 bp was obtained, which is species specific for DCA120 (**Figure 5**) and other Indian senna accessions. With CBDP-25, signature amplicons of approximately 575 and 345 bp were obtained, which are species specific for DCA13 (**Figure 6**) and DCA119 (**Figure 7**), respectively, and other Indian senna accessions.

### 3.3 Sequence analysis of SCAR markers

Primers for SCAR (both SCoT and CBDP based) markers were designed in line with the standard conventions of primer design such as ensuring maintenance of GC content of the primers at 50% and then further ensuring that the primers end either with a G or C. The SCAR sequences specific for the accessions of Indian senna were deposited in GenBank, and accession numbers were obtained for these SCoT- and CBDP-based SCARs. Based on these sequences, SCoT–SCAR (**Figure 5**, GenBank accession no. OR060948) and CBDP–SCARs (**Figure 6**, GenBank accession no. OR060949 and **Figure 7** GenBank accession no. OR060950) primer pairs were designed. Based on these sequences, a SCoT–SCAR primer pair, CA120SSF2 (5′ACGACATGGCGACCCACACCCGGTG3′), CA120SSR2 (3′ACGACATGGCGACCCACAATGGAACTGGG5′) and two CBDP–SCAR primer pairs were designed: CA13CSF1 (CTGAGCACGATCCAATGTATGCTTACAAGTC), CA13CSR1 (3′CTGAGCACGATCCAATGTTCAGCACACAATCC5′) and CA119CSF2 (5′CTGAGCACGATCCAATGTTCAGCACACAATCC3′), CA119CSF2 (3′CTGAGCACGATCCATAGTATATAGCTATGGAATTGTCC5′), respectively (**Table 5**).

**Table 5:** Details of SCAR primers developed from SCoT-44 and CBDP-25 for Indian senna.

Primary Primer	SCAR Primer	SCAR primer Sequence (5’-3’)	Length (bases)	Annealing temperature (°C)	Amplicon Length (bp)	GenBank Accession No.
SCoT-44	CA120SSF2 CA120SSR2	ACGACATGGCGACCCACACCCGGTG ACGACATGGCGACCCACAATGGAACTGGG	25 29	64°C	287	OR060948
CBDP-25	CA13CSF1 CA13CSR1 CA119CSF2 CA119CSR2	CTGAGCACGATCCAATGTATGCTTACAAGTC CTGAGCACGATCCAATGTTCAGCACACAATCC CTGAGCACGATCCAATGTTCAGCACACAATCC CTGAGCACGATCCATAGTATATAGCTATGGAATTGTCC	31 32 32 38	64.5°C 64.5°C	575 345	OR060949 OR060950

### 3.4 Validation of developed SCAR markers by PCR

**Figure 8:**
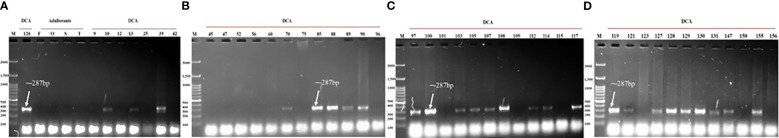
Validation of the newly designed SCoT-44 SCAR across panel of DCA120 and other Indian senna accessions along with the four adulterant species. Lane M—100-bp DNA ladder. **(A)** DCA120, *Cassia fistula* (F), *Senna occidentalis* (O), *Senna sophera* (S), *Senna tora* (T), DCA09–DCA42 accessions; **(B)** DCA45–DCA96; **(C)** DCA97–DCA117; **(D)** DCA119–DCA156.

**Figure 9:**
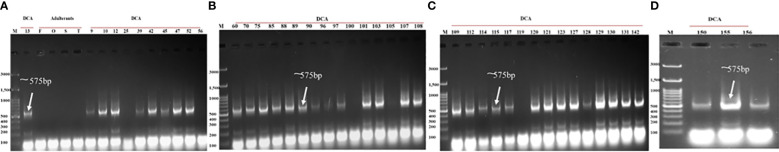
Validation of the newly designed CBDP-25 SCAR across panel of DCA13 and other accessions of Indian senna along with the four adulterant species. Lane M—100-bp DNA ladder. **(A)** DCA13, *Cassia fistula* (F), *Senna occidentalis* (O), *Senna sophera* (S), *Senna tora* (T), DCA09–DCA60 accessions; **(B)** DCA70–DCA109; **(C)** DCA112–DCA142; **(D)** DCA150–DCA156.

**Figure 10:**
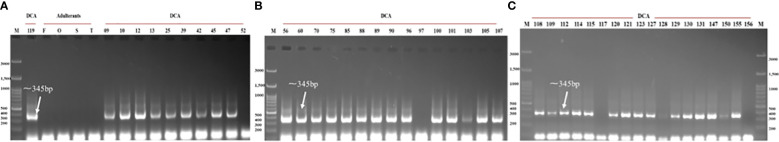
Validation of the newly designed CBDP-25 SCAR across panel of DCA119 and other Indian senna accessions and the four adulterant species. Lane M - 100-bp DNA ladder. **(A)** DCA119, *Cassia fistula* (F), *Senna occidentalis* (O), *Senna sophera* (S), *Senna tora* (T), DCA09–DCA52; **(B)** DCA56–DCA107; **(C)** DCA108–DCA156.

All 44 samples of Indian senna and its 4 adulterant species were amplified using the developed SCAR primers. SCAR was standardized at an annealing temperature of 64°C in the case of the SCoT-44 SCAR marker (**Figure 8**). In the case of the CBDP-25 SCAR marker, annealing temperature of 64.5°C was standardized for DCA13 (**Figure 9**) and DCA119 (**Figure 10**). All the developed and validated SCoT- and CBDP-based SCAR markers are specific to the accessions of Indian senna but not to the other four species (**Table 5**).

## 4 Discussion

Authentication of true-to-type medicinal plants is the imminent need of the hour to preserve the uniqueness and individuality of the plant under question. This exercise of authentication has been fairly facilitated using various techniques such as morphological and biochemical analysis (Bekbolatova et al., 2018; Seethapathy et al., 2018), DNA barcoding (Hebert et al., 2003; Mishra et al., 2016), DNA barcoding coupled with HRM curve analysis (Mishra et al., 2018), and molecular markers (Collard and Mackill, 2009). Approaches, such as powder microscopy, enhance taxonomic recognition of a particular genus by projecting micro morphological and anatomical characters (Nesmerak et al., 2020). But these characters need not be unique and may lead to spurious identification and authentication of a species and thus diminish the very purpose of employing the same. Biochemical approach of identification and authentication of a species is very much influenced by age, physiological condition, and environmental factors (Chan, 2003). DNA-based markers have proven to be the best for deciphering authenticity and genetic diversity among plants as they are highly discriminatory, environmental neutral, are more objective and reliable, and unlimited in number. DNA barcoding in conjunction with HRM curve analysis has been used for undisputed authentication of Indian senna (Mishra et al., 2018). Gene-based markers, such as SCoT, proved to be superior over non-genic markers, such as RAPD and ISSR, in terms of diversity index, marker index, and resolving power. SCoT yielded more polymorphism and scorable amplicons compared to RAPD in tetraploid potato (Gorji et al., 2011). SCoT is highly informative with pronounced discriminating power than RAPD and ISSR as reported for bamboo (Amom et al., 2020).

SCoT and CBDP marker-based studies have been used for the discrimination of genuine and adulterant samples of crop plants. Both SCoT and CBDP markers use of a single primer, which acts as both forward and reverse. Further, the polymorphism, which arises based on their application, is essentially either in the coding region (SCoT) or regulatory region such as a promoter (CBDP). The polymorphic amplicons act as markers and have a pivotal role in species authentication. However, the use of a single primer in SCoT and CBDP PCR results in a multitude of amplicons across the panel of accessions being investigated, thus generating profiles which could be non-reproducible. This drawback of the non-reproducibility and multi-locus nature of SCoT and CBDP markers is resolved by cloning, sequencing, and extending the length of the initial primer used and thus generating a single-locus sequence-specific signature SCAR. SCAR is a secondary, sequence-specific (either co-dominant or dominant) marker (Paran and Michelmore, 1993).

In the present study, efforts were made to develop SCoT- and CBDP-based SCAR markers for the identification of Indian senna to facilitate its fool-proof authentication. Forty-four accessions of Indian senna and four adulterant species were included in the study. The work reports the development of highly sensitive, effective, and reproducible PCR-based SCoT–SCARs and CBDP–SCARs for identifying true-to-type Indian senna. SCAR markers have been developed for the authentication of apple (De Clercq et al., 2003), papaya (Deputy et al., 2002), rose (Bashir et al., 2014), cowpea (Boukar et al., 2004), strawberry (Rugienius et al., 2006), buffelgrass (Dwivedi et al., 2007), and brassica (Piquemal et al., 2005). SCAR markers have been used for molecular verification of medicinal plant species such as Ashoka (*Saraca asoca* (Roxb.) de Wild) and its adulterant *Polyalthia longifolia* Benth (Kumar, 2016), *Artemisia*, *Phyllanthus*, *Panax* (Kiran et al., 2010), and *Dimocarpus longan* varieties (Yang et al., 2013). Germplasm and cultivar-specific SCoT-based SCAR was identified in *Dendrobium officinale* (Zheng et al., 2021) and *Chrysanthemum morifolium* Ramat (Cai et al., 2022). Deng (2021) developed SCoT-based SCAR to detect smut resistance in sugarcane.

Parallel to this, CBDP-based SCAR markers have been developed in the present study. Deciphering the true identity of an individual plant species can be facilitated using CBDP-derived markers as they are species neutral (Singh et al., 2014). Cost effectiveness, high polymorphism, high reproducibility, and detailed genetic information can be made available using the CBDP marker system (Etminan et al., 2018). The present study is the first of its kind to use CBDP-based SCARs for the authentication of Indian senna.

A cloning-based RAPD–SCAR marker was developed by Yang et al. (2013) to distinguish *D. longan* from *Dimocarpus confinis*. Cheng et al. (2016) developed a SCAR marker to authenticate litchi species and resolved issues pertaining to naming and identification of several litchi cultivars grown internationally. Jose et al. (2021) developed an ISSR-based SCAR marker toward the identification of cardamom Malabar (prostrate panicle) variety (*Elettaria cardamomum* L. Maton). Using ISSR, SCoT and CBDP markers, the genetic diversity of Iranian *Aegilops triuncialis* accessions was evaluated by Khodaee et al. (2021). Singh et al. (2014) validated the utility of the CBDP marker across cultivars of cotton (*Gossypium* species), jute (*Corchorus capsularis* and *Corchorus olitorius*), and linseed (*Linum usitatissimum*).

All these reported findings further strengthen the application of SCoT and CBDP either singly or in combination with SCAR, which takes it to a further higher level of discriminating the true-to-type species.

The diagnostic markers developed in the present study facilitate identification and authentication of true-to-type Indian senna and intend to unambiguously resolve the issue of poor quality control, and thus help in sustainable exploitation of Indian senna.

## 5 Conclusion

Starting from multi-locus, PCR-based markers, such as SCoT and CBDP, single locus, highly reproducible, sequence-specific SCAR markers have been developed in the present study to resolve the identity of true-to-type Indian senna accessions against the four adulterant species. In this study, one SCoT-44 SCAR species-specific primer pair (CA120SSF2/CA120SSR2) and two CBDP-25 SCAR species-specific primer pairs (CA13CSF1/CA13CSR1 and CA119CSF2/CA119CSR2) have been developed for Indian senna. The SCoT- and CBDP-derived SCAR markers developed in the present study can be seen as a translational tool to wedge the distance between the lab and field and facilitate rapid, unequivocal, and effective identification of Indian senna paving way for its conservation and sustainable utilization.

## Data availability statement

The datasets presented in this study can be found in online repositories. The names of the repository/repositories and accession number(s) can be found below: https://www.ncbi.nlm.nih.gov/genbank/, OR060948; https://www.ncbi.nlm.nih.gov/genbank/, OR060949; https://www.ncbi.nlm.nih.gov/genbank/, OR060950.

## Author contributions

SC: Data curation, Formal analysis, Investigation, Methodology, Software, Validation, Visualization, Writing – original draft, Writing – review & editing. MA: Data curation, Formal analysis, Investigation, Methodology, Software, Validation, Visualization, Writing – original draft, Writing – review & editing, Conceptualization, Funding acquisition, Project administration, Resources, Supervision. PK: Data curation, Formal analysis, Investigation, Methodology, Software, Validation, Visualization, Writing – original draft. SF: Data curation, Formal analysis, Investigation, Methodology, Visualization, Writing – original draft. MP: Formal analysis, Resources, Software, Visualization, Writing – original draft. CK: Conceptualization, Formal analysis, Visualization, Writing – review & editing. RNR: Formal analysis, Resources, Visualization, Writing – original draft.
